# Association of stress hyperglycemia ratio with presence and severity of chronic kidney disease among US adults with diabetes mellitus

**DOI:** 10.3389/fendo.2024.1446390

**Published:** 2024-10-22

**Authors:** Wenguang Lai, Yaxin Meng, Yang Zhou, Tingting Zhang, Baoyuan Zhang, Zhidong Huang, Zhiyong Gao

**Affiliations:** ^1^ Heyuan People’s Hospital, Guangdong Provincial People’s Hospital, Heyuan Hospital, Heyuan, China; ^2^ School of Foreign Studies, Southern Medical University, Guangzhou, Guangdong, China; ^3^ College of Pharmacy, Guangdong Pharmaceutical University, Guangzhou, China

**Keywords:** stress hyperglycemia ratio, diabetes mellitus, chronic kidney disease, chronic kidney disease severity, adverse renal outcomes

## Abstract

**Background:**

Among diabetes mellitus (DM) patients, stress hyperglycemia ratio (SHR) is a strong predictor of short- and long-term prognosis, and adverse cardiovascular events. However, whether SHR is associated with increased risk of presence and severity of chronic kidney (CKD) disease remains undetermined.

**Methods:**

Patients with DM from the National Health and Nutrition Examination Survey (NHANES) database (1999–2020) were included and divided into 5 groups according to their SHR level (quintile 1 to 5). Study outcomes were CKD, advanced CKD (ACKD), and CKD severity. Logistic regression and restricted cubic spline (RCS) were used to assess the association between the SHR and outcomes.

**Results:**

Totally, 6,119 patients were included. After adjustment, compared to patients with SHR in quintile 3 (as reference), the risk of CKD is 1.50 (P<0.001) for quintile 1, 1.23 (P=0.140) for quintile 2, 1.95 (P<0.001) for quintile 4, and 1.79 (P<0.001) for quintile 5. For the risk of ACKD, the OR is 1.46 (P=0.410) for quintile 1, 1.07 (P=0.890) for quintile 2, 3.28 (P=0.030) for quintile 4, and 3.89 (P=0.002) for quintile 5. For the CKD severity, the OR is 1.46 (P<0.001) for quintile 1, 1.20 (P=0.163) for quintile 2, 1.84 (P<0.001) for quintile 4, and 1.83 (P<0.001) for quintile 5. RCS analysis also showed a U-shaped association between SHR and outcomes (All P for nonlinearity<0.05).

**Conclusion:**

Our study demonstrated that too low or too high SHR level is significantly associated with adverse renal outcomes in patients with DM.

## Introduction

Diabetes mellitus (DM) is a serious chronic disease, becoming the 7th leading cause of mortality in the United States (US), and it is predicted that about 1/3 of the population in the US will suffer from DM by 2025 (1–3). The hyperglycemic status of individuals with DM may further lead to a higher prevalence of diseases related to chronic inflammation.

Chronic kidney disease (CKD) is a common and morbid comorbidity of DM, and hyperglycemia is one of its main causes (4). Over 40% of persons with DM occur the deterioration of kidney function, manifested as albuminuria or impaired glomerular filtration rate, and further develop into advanced CKD (ACKD) (5–7). Although current treatment options for CKD are varied (including renin-angiotensin system inhibitors [RASi], sodium-glucose cotransporter-2 inhibitors, and glucagon-like peptide-1 agonists), however, for ACKD patients, such treatment can only delay the onset of the disease and increase the patients’ healthcare costs (8–12). Therefore, early detection and identification of high-risk patients and their risk factors are essential to moderate the progression of CKD and reduce its morbidity and mortality in patients with DM.

Stress hyperglycemia refers to a temporary rise in blood glucose caused by physiological or psychological stress, and in order to better reflect this state of patients, the stress hyperglycemia ratio (SHR) index has been proposed (13). Currently, SHR is reported to be associated with poor prognosis in high-risk patients with acute myocardial infarction (AMI), ischemic stroke, or critically ill patients (14–18). In addition, previous studies have found SHR is a better predictor of acute kidney injury among patients with cardiac arrest and AMI (19, 20). In community general DM population, SHR also had a good predictive value for all-cause and cardiovascular mortality (21, 22). However, whether SHR can be a good predictor for CKD progression and severity has not been clearly investigated in DM patients.

Therefore, this study was designed to assess the relationship between SHR and adverse renal outcome (CKD, ACKD, and CKD severity) among DM patients in a large, nationally representative population in the US.

## Methods

### Study design and population

We employed data from the National Health and Nutrition Examination Survey (NHANES) database, a program administered by the Centers for Disease Control and Prevention and the National Centers for Health Statistics in the US. Overall, 107,622 participants were covered during 1999–2020.

**Figure 1:**
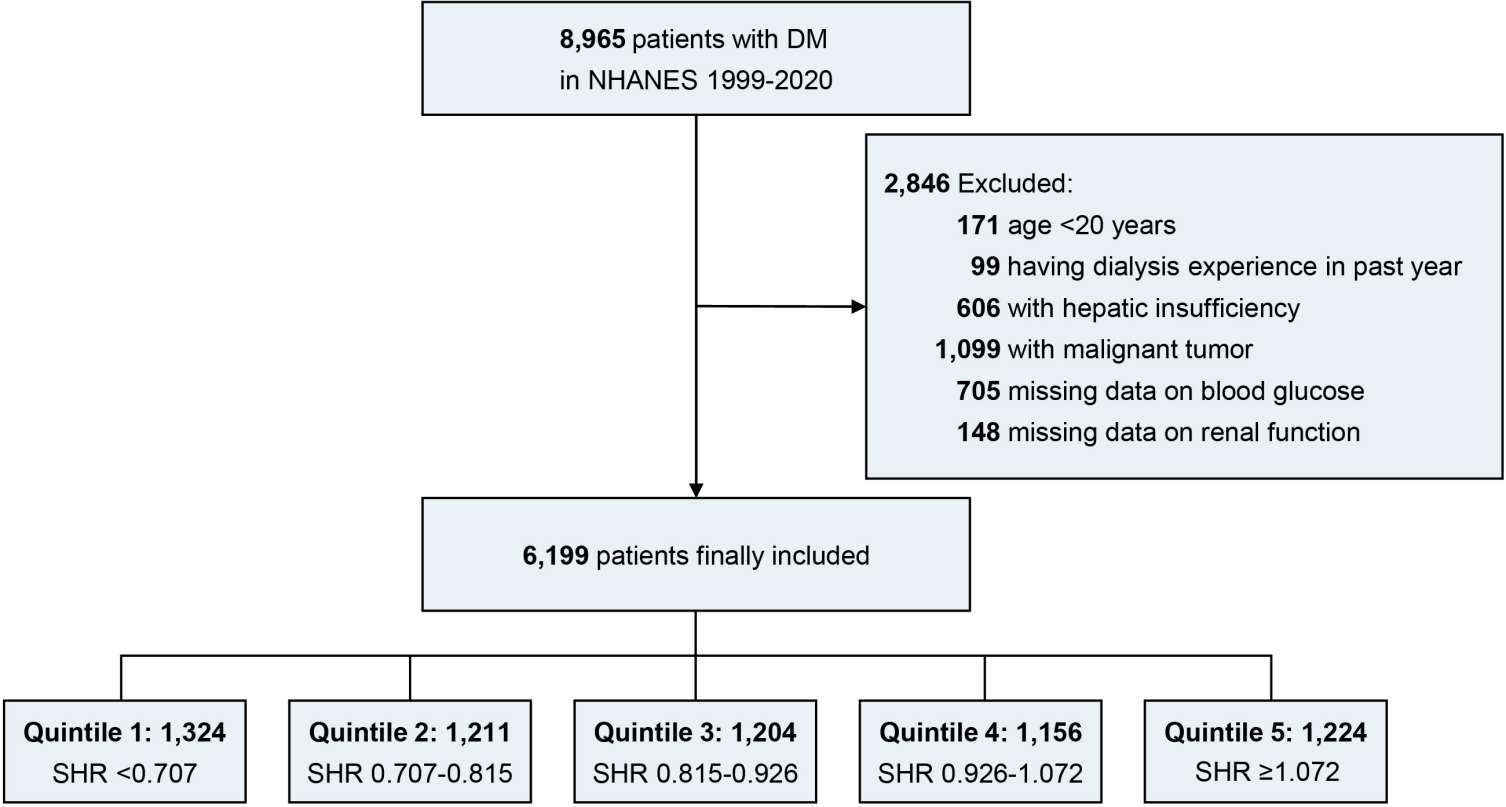
Flow diagram. DM, diabetes mellitus; NHANES, national health and nutrition examination survey; SHR, stress hyperglycemia ratio.

Patients meeting the following criteria were included (1): combined with DM (2); age over 20 years. Patients meeting the following criteria were excluded (1): having dialysis experience in the past year (2); combined with hepatic insufficiency (3); combined with malignant tumor (4); insufficient or missing data on blood glucose (random blood glucose [RBG] or glycated hemoglobin [HbA1c]) (5); insufficient or missing data on renal function (estimated glomerular filtration rate [eGFR] and albuminuria). Finally, a total of 6,119 eligible patients with DM were included in the study analysis (**Figure 1**).

### Outcomes and exposure definitions

The primary outcomes were CKD, ACKD, and CKD severity. The secondary outcomes were eGFR categories and albuminuria categories (**Supplementary Table S1**, **Supplementary Figure S1**). SHR was calculated by the formula: SHR = RBG (mmol/L)/(1.59 * HbA1c [%] – 2.59) (23). DM is determined according to patients with HbA1c ≥ 6.5% or response ‘yes’ to the question: ‘Doctor told you have diabetes’ or ‘Taking insulin now’ (22, 24). The eGFR categories were G1 (eGFR>90 ml/min/1.73 m^2^), G2 (eGFR 60-89 ml/min/1.73 m^2^), G3a (eGFR 45-59 ml/min/1.73 m^2^), G3b (eGFR 30-44 ml/min/1.73 m^2^), G4 (eGFR 15-29 ml/min/1.73 m^2^) and G5 (eGFR <15 ml/min/1.73 m^2^). Albuminuria categories were A1 (uACR<30 mg/g), A2 (uACR 30-300 mg/g) and A3 (uACR >300 mg/g). According to eGFR categories and albuminuria categories, CKD severity was divided into low risk (G1, G2, and A1), moderate risk ([G3a and A1] or [G1, G2, and A2]), high risk ([G3b and A1], [G3a and A2], or [G1, G2, and A3]) and very high risk ([G4, G5, and A1], [G3b, G4, G5, and A2], or [G3a, G3b, G4, G5 and A3]) (25). CKD was defined as eGFR ≤ 60 ml/min/1.73 m^2^ or uACR ≥30 mg/g. ACKD was defined as eGFR ≤ 30 ml/min/1.73 m^2^.

### Covariates definitions

Baseline information regarding age, gender, race, education, smoking status, alcohol consumption, and medical history (including cardiovascular disease, hypertension, DM duration, and family history of DM, was collected through self-reported questionnaires. Individuals were classified based on their smoking history: never smoked (consumed less than 100 cigarettes in their lifetime), former smokers (smoked over 100 cigarettes but had quit by the survey date), and current smokers (smoked at least 100 cigarettes and still smoked at the survey time). Alcohol intake was divided into none (0 g/day), moderate (0.1 to 27.9 g/day for men and 0.1 to 13.9 g/day for women), and heavy (≥28 g/day for men and ≥14 g/day for women). Cardiovascular disease is composed of heart attack, congestive heart failure, angina, coronary heart disease, and stroke. Anemia is defined as men with a hemoglobin level less than 130 g/L and women with a hemoglobin level less than 120 g/L according to World Health Organization standards (26).

Further details can be found at https://www.cdc.gov/nchs/nhanes/index.htm.

### Statistical analysis

Since 1999, the NHANES database has been released in 2-year cycles, and the 2017-March 2020 prepandemic data represent a 3.2-year period (a total of 21.2 years). New multicycle sample weights were calculated based on the sample weights of the combined survey cycles with the following formulas:

In 1999-2002, the examination weight = (4/21.2) × WTMEC4YR;

In 2003-2016, the examination weight = (2/21.2) × WTMEC2YR;

In 2017-2020, the examination weight = (3.2/21.2) × WTMECPRP (27).

All statistical analyses employed complex sample weighting analysis utilizing the recommended NHANES interview weights (R package “survey”).

Patients were divided into 5 groups based on the quartile of SHR level. Continuous data were shown as mean ( ± SE) for normally distributed data and median (IQR) for non-normally distributed data, and categorical data were presented as counts and weighted percentages. ANOVA analysis, the Kruskal-Wallis test for continuous variables, or the chi-square test for categorical data, as applicable, were used to compare the groups.

Multivariable logistic regression models were performed to assess the relationship between SHR and CKD or ACKD. Ordinal logistic regression models were used to investigate the relationship between SHR and CKD severity. Characteristic variables with significant baseline differences or clinical significance were included in multivariable regression models, and we included age, gender, race, body mass index (BMI), smoking status, alcohol consumption, DM duration, hypertension, cardiovascular disease (CVD), anemia, uric acid, blood urea nitrogen, antidiabetic drugs, RASi, and statins in the final model. In addition, restricted cubic spline (RCS) was used to explore the association between SHR and outcomes, and the value of SHR at the lowest risk was selected as the cutoff value. Furthermore, subgroup analyses were also performed to assess the influence of SHR on CKD severity in different subgroups stratified by gender (male and female), age (<60 and ≥60 years), smoking status (ex-smoker and never smoked), BMI level (<30 and ≥30), CVD (with and without), and hypertension (with and without), using generalized linear regression models. The statistical analysis was performed by R software (version 4.2.1). A two-tailed P value < 0.05 was considered statistically significant.

## Results

We included 6,119 DM patients (mean age [SE]: 57.64 ± 0.27 years; 47.87% female) in study analysis, and patients were divided into 5 groups based on their SHR level (quintile 1 [n= 1324], SHR < 0.707; quintile 2 [n= 1211], 0.707 ≤ SHR < 0.815; quintile 3 [n= 1204], 0.815 ≤ SHR <0.926; quintile 4 [n=1156], 0.926 ≤ SHR <1.072; quintile 5 [n=1224], SHR≥ 1.072). Totally 17.37% of patients (n=1040) were current smokers, 8.10% of patients (n=428) were heavy drinkers, and the mean BMI level of the patients was 33.05 kg/m^2^. In addition, 61.34% of patients (n=3,865) had hypertension, 22.39% of patients (n=1,457) had CVD and 12.62% of patients (n=1,000) had anemia. The mean eGFR level was 86.32 ± 0.44 ml/min/1.73 m^2^, and the median urinary albumin creatinine ratio level was 11.96 [6.39-36.81] mg/g (**Table 1**).

**Table 1:** Baseline characteristics between groups stratified by quintile of SHR.

Characteristics	Overall N=6,119	Quintile 1 SHR <0.707 N=1,324	Quintile 2 0.707-0.815 N=1,211	Quintile 3 0.815-0.926 N=1,204	Quintile 4 0.926-1.072 N=1,156	Quintile 5 SHR ≥1.072 N=1,224	P value
SHR level	0.90 ± 0.00	0.59 ± 0.00	0.76 ± 0.00	0.87 ± 0.00	0.99 ± 0.00	1.29 ± 0.01	<0.001
Age, years	57.64 ± 0.27	58.04 ± 0.50	57.96 ± 0.51	57.84 ± 0.57	57.06 ± 0.61	57.29 ± 0.52	0.569
Female	2946 (47.87)	667 (51.25)	633 (52.13)	592 (50.39)	530 (43.64)	524 (41.92)	<0.001
Body mass index, kg.m^2^	33.05 ± 0.16	32.92 ± 0.28	33.45 ± 0.30	33.11 ± 0.31	33.23 ± 0.33	32.52 ± 0.30	0.208
Race							<0.001
Hispanic	1935 (17.60)	390 (16.67)	338 (14.97)	392 (17.94)	394 (18.64)	421 (19.80)	
Non-hispanic black	1706 (16.53)	455 (21.75)	399 (19.49)	299 (14.45)	271 (13.26)	282 (13.68)	
Non-hispanic white	1862 (56.18)	354 (51.61)	345 (55.41)	375 (56.36)	376 (58.62)	412 (58.93)	
Other	616 (9.68)	125 (9.96)	129 (10.13)	138 (11.25)	115 (9.49)	109 (7.59)	
Smoking status							0.321
Never	3146 (50.63)	707 (52.60)	658 (52.99)	599 (49.22)	583 (50.20)	599 (48.31)	
Former	1927 (31.93)	392 (28.90)	354 (31.48)	379 (31.65)	374 (32.16)	428 (35.58)	
Current	1040 (17.37)	224 (18.50)	197 (15.54)	225 (19.13)	199 (17.64)	195 (16.10)	
Alcohol consumption							0.672
None	4904 (79.42)	1085 (84.40)	957 (84.64)	998 (85.93)	919 (83.26)	945 (82.01)	
Moderate	398 (6.95)	87 (8.01)	83 (6.97)	72 (5.71)	82 (8.25)	74 (7.90)	
Heavy	428 (8.10)	72 (7.59)	84 (8.40)	81 (8.36)	88 (8.48)	103 (10.09)	
Medical history							
Hypertension	3865 (61.34)	843 (62.09)	790 (64.38)	769 (62.45)	714 (59.89)	749 (58.71)	0.290
Cardiovascular disease	1457 (22.39)	308 (21.80)	269 (19.68)	292 (22.92)	255 (21.37)	333 (26.19)	0.071
Anemia	1000 (12.62)	264 (15.83)	193 (12.45)	180 (11.53)	162 (11.33)	201 (12.02)	0.052
Family history of DM	4120 (67.61)	903 (70.29)	798 (67.66)	814 (70.29)	755 (67.48)	850 (70.98)	0.560
DM duration							<0.001
<5 years	1431 (25.67)	286 (23.10)	306 (29.13)	319 (28.71)	283 (25.88)	237 (22.06)	
6-9 years	1031 (17.31)	208 (14.79)	203 (15.98)	205 (18.60)	189 (18.15)	226 (19.40)	
10-19 years	1340 (21.51)	294 (22.66)	221 (17.73)	235 (19.62)	276 (22.02)	314 (25.94)	
≥20 years	989 (15.09)	247 (19.77)	159 (11.09)	172 (12.15)	166 (14.81)	245 (17.90)	
Missed diagnosis	1287 (20.02)	280 (19.68)	316 (26.06)	267 (20.92)	231 (19.13)	193 (14.71)	
Laboratory indexes							
HbA1c, %	7.48 ± 0.03	7.45 ± 0.06	7.09 ± 0.05	7.13 ± 0.07	7.61 ± 0.07	8.12 ± 0.09	<0.001
Random blood glucose, mg/dL	153.21 ± 1.17	98.11 ± 1.08	119.76 ± 1.23	137.29 ± 1.73	170.64 ± 2.21	240.36 ± 3.28	<0.001
eGFR, mL/min/1.73m^2^	86.32 ± 0.43	84.62 ± 0.97	86.21 ± 0.85	87.65 ± 0.76	87.48 ± 1.04	85.63 ± 0.95	0.088
uACR, mg/g	11.96 (6.34,36.81)	11.71 (6.02,35.04)	10.57 (5.99,30.11)	10.54 (5.90,25.07)	13.81 (6.55,43.11)	16.60 (6.97,55.56)	<0.001^a^
White blood cell, 10^9/L	7.83 ± 0.04	8.13 ± 0.10	7.85 ± 0.09	7.61 ± 0.09	7.74 ± 0.09	7.83 ± 0.08	0.004
Lymphocyte, 10^9/L	2.24 ± 0.02	2.46 ± 0.05	2.30 ± 0.04	2.22 ± 0.04	2.14 ± 0.03	2.08 ± 0.03	<0.001
Neutrophile, 10^9/L	4.72 ± 0.03	4.75 ± 0.07	4.67 ± 0.07	4.53 ± 0.06	4.75 ± 0.07	4.92 ± 0.06	<0.001
Blood urea nitrogen, mmol/L	5.67 ± 0.05	5.73 ± 0.09	5.60 ± 0.10	5.47 ± 0.08	5.60 ± 0.09	5.94 ± 0.13	0.036
Uric acid, μmol/L	336.39 ± 1.93	336.35 ± 3.91	344.49 ± 3.92	338.79 ± 3.66	336.59 ± 4.58	325.70 ± 3.96	0.008
Medication							
Antidiabetic drug	4132 (68.01)	944 (73.17)	752 (61.39)	760 (64.71)	782 (68.28)	894 (72.99)	<0.001
RASi	3083 (49.33)	685 (51.69)	613 (50.30)	651 (53.34)	541 (46.36)	593 (45.38)	0.033
Statins	2653 (44.60)	585 (44.22)	539 (46.23)	521 (44.90)	484 (42.85)	524 (45.14)	0.762

Data are means ± SD, median (interquartile range), or n (%).

DM, diabetes mellitus; eGFR, estimated glomerular filtration rate; HbA1c, glycated hemoglobin; SHR, stress hyperglycemia ratio; RASi, renin angiotensin system inhibitor; uACR, urea albumin creatinine ratio.

a uACR employed the Kruskal-Wallis test; ANOVA analysis for other continuous variables; and the chi-square test for categorical variables.

With CKD severity, patients were older (low to very risk = 54.92: 59.65: 64.91: 69.69 years, P<0.001), had higher SHR levels (low to very risk = 0.89: 0.90: 0.93: 0.95, P=0.003), higher prevalence of hypertension (low to very risk = 55.74%: 66.32%: 78.25%: 82.44%, P<0.001), cardiovascular disease (low to very risk = 16.57%: 25.93%: 37.31%: 52.89%, P<0.001), and anemia (low to very risk = 8.96%: 11.54%: 22.25%: 46.75%, P<0.001) (**Table 2**).

**Table 2:** Baseline characteristics between groups stratified by CKD severity.

Characteristics	Overall N=6,119	Low risk N=3,625	Moderate risk N=1,495	High risk N=607	Very high risk N=392	P value
SHR level	0.90 ± 0.00	0.89 ± 0.01	0.90 ± 0.01	0.93 ± 0.01	0.95 ± 0.02	0.003
Age (years)	57.64 ± 0.27	54.92 ± 0.29	59.65 ± 0.61	64.91 ± 0.71	69.69 ± 0.77	<0.001
Female	2946 (47.87)	1788 (48.48)	699 (45.45)	276 (46.13)	183 (54.38)	0.130
Body mass index, kg.m^2^	33.05 ± 0.16	32.98 ± 0.19	33.24 ± 0.30	33.00 ± 0.47	33.08 ± 0.46	0.871
Race						0.602
Hispanic	1935 (17.60)	1192 (17.76)	471 (18.74)	173 (15.97)	99 (13.29)	
Non-hispanic black	1706 (16.53)	1002 (16.11)	423 (17.02)	160 (16.67)	121 (19.22)	
Non-hispanic white	1862 (56.18)	1045 (56.46)	459 (54.25)	220 (58.22)	138 (58.17)	
Other	616 (9.68)	386 (9.67)	142 (9.99)	54 (9.14)	34 (9.33)	
Smoking status						<0.001
Never	3146 (50.63)	1935 (52.80)	718 (46.46)	306 (48.74)	187 (46.75)	
Former	1927 (31.93)	1053 (29.04)	497 (34.89)	212 (38.30)	165 (43.81)	
Current	1040 (17.37)	634 (18.17)	279 (18.65)	87 (12.95)	40 (9.44)	
Alcohol consumption						0.061
None	4904 (79.42)	2892 (82.88)	1190 (84.73)	493 (86.90)	329 (91.02)	
Moderate	398 (6.95)	243 (7.68)	94 (7.07)	37 (6.15)	24 (6.75)	
Heavy	428 (8.10)	275 (9.44)	109 (8.20)	35 (6.96)	9 (2.23)	
Medical history						
Hypertension	3865 (61.34)	2083 (55.74)	995 (66.32)	464 (78.25)	323 (82.44)	<0.001
Cardiovascular disease	1457 (22.39)	609 (16.57)	422 (25.93)	222 (37.31)	204 (52.89)	<0.001
Anemia	1000 (12.62)	392 (8.96)	250 (11.54)	153 (22.25)	205 (46.75)	<0.001
Family history of DM	4120 (67.61)	2458 (69.83)	1002 (70.35)	399 (65.85)	261 (64.68)	0.230
DM duration						<0.001
<5 years	1431 (25.67)	992 (29.57)	318 (23.44)	90 (14.30)	31 (8.59)	
6-9 years	1031 (17.31)	622 (17.47)	253 (17.38)	107 (18.94)	49 (13.67)	
10-19 years	1340 (21.51)	707 (19.20)	369 (24.94)	141 (23.79)	123 (32.43)	
≥20 years	989 (15.09)	414 (11.40)	260 (15.79)	162 (27.13)	153 (38.68)	
Missed diagnosis	1287 (20.02)	876 (22.36)	283 (18.45)	98 (15.84)	30 (6.63)	
Laboratory indexes						
HbA1c, %	7.48 ± 0.03	7.31 ± 0.04	7.75 ± 0.06	8.01 ± 0.11	7.56 ± 0.09	<0.001
Random blood glucose, mg/dL	153.21 ± 1.17	146.94 ± 1.42	160.59 ± 2.47	173.04 ± 4.44	163.22 ± 4.81	<0.001
eGFR, mL/min/1.73m^2^	86.32 ± 0.43	94.12 ± 0.37	83.60 ± 1.00	66.10 ± 1.50	36.57 ± 0.82	<0.001
uACR, mg/g	11.96 (6.34,36.81)	8.00 (5.32,13.18)	46.19 (24.10,90.25)	147.57 (29.00,681.12)	341.18 (65.31,1086.02)	<0.001^a^
White blood cell, 10^9/L	7.83 ± 0.04	7.75 ± 0.05	7.92 ± 0.09	8.20 ± 0.14	7.89 ± 0.14	0.022
Lymphocyte, 10^9/L	2.24 ± 0.02	2.27 ± 0.02	2.24 ± 0.05	2.21 ± 0.07	1.98 ± 0.06	<0.001
Neutrophile, 10^9/L	4.72 ± 0.03	4.63 ± 0.04	4.80 ± 0.06	5.03 ± 0.10	4.95 ± 0.12	<0.001
Blood urea nitrogen, mmol/L	5.67 ± 0.05	4.98 ± 0.04	5.61 ± 0.08	7.62 ± 0.18	11.20 ± 0.34	<0.001
Uric acid, μmol/L	336.39 ± 1.93	318.23 ± 2.15	345.78 ± 3.49	387.26 ± 6.40	431.65 ± 7.47	<0.001
Medication						
Antidiabetic drug	4132 (68.01)	2303 (65.11)	1054 (70.63)	451 (73.15)	324 (85.21)	<0.001
RASi	3083 (49.33)	1678 (44.94)	810 (54.67)	363 (62.17)	232 (59.08)	<0.001
Statins	2653 (44.60)	1466 (43.29)	645 (41.85)	312 (52.97)	230 (60.59)	<0.001

Data are means ± SD, median (interquartile range), or n (%).

CKD severity: G1: eGFR>90 ml/min/1.73 m^2^, G2: eGFR 60-89 ml/min/1.73 m^2^, G3a: eGFR 45-59 ml/min/1.73 m^2^, G3b: eGFR 30-44 ml/min/1.73 m^2^, G4: eGFR 15-29 ml/min/1.73 m^2^, G5: eGFR <15 ml/min/1.73 m^2^; A1: uACR<30 mg/g, A2: uACR 30-300 mg/g, A3: uACR >300 mg/g; low risk: G1, G2, and A1, moderate risk: [G3a and A1] or [G1, G2 and A2], high risk: [G3b and A1], [G3a and A2], or [G1, G2 and A3], very high risk: [G4, G5 and A1], [G3b, G4, G5 and A2], or [G3a, G3b, G4, G5 and A3].

DM, diabetes mellitus; eGFR, estimated glomerular filtration rate; HbA1c, glycated hemoglobin; SHR, stress hyperglycemia ratio; RASi, renin angiotensin system inhibitor; uACR, urea albumin creatinine ratio.

a uACR employed the Kruskal-Wallis test; ANOVA analysis for other continuous variables; and the chi-square test for categorical variables.

### Association between SHR and adverse renal outcomes

**Figure 2:**
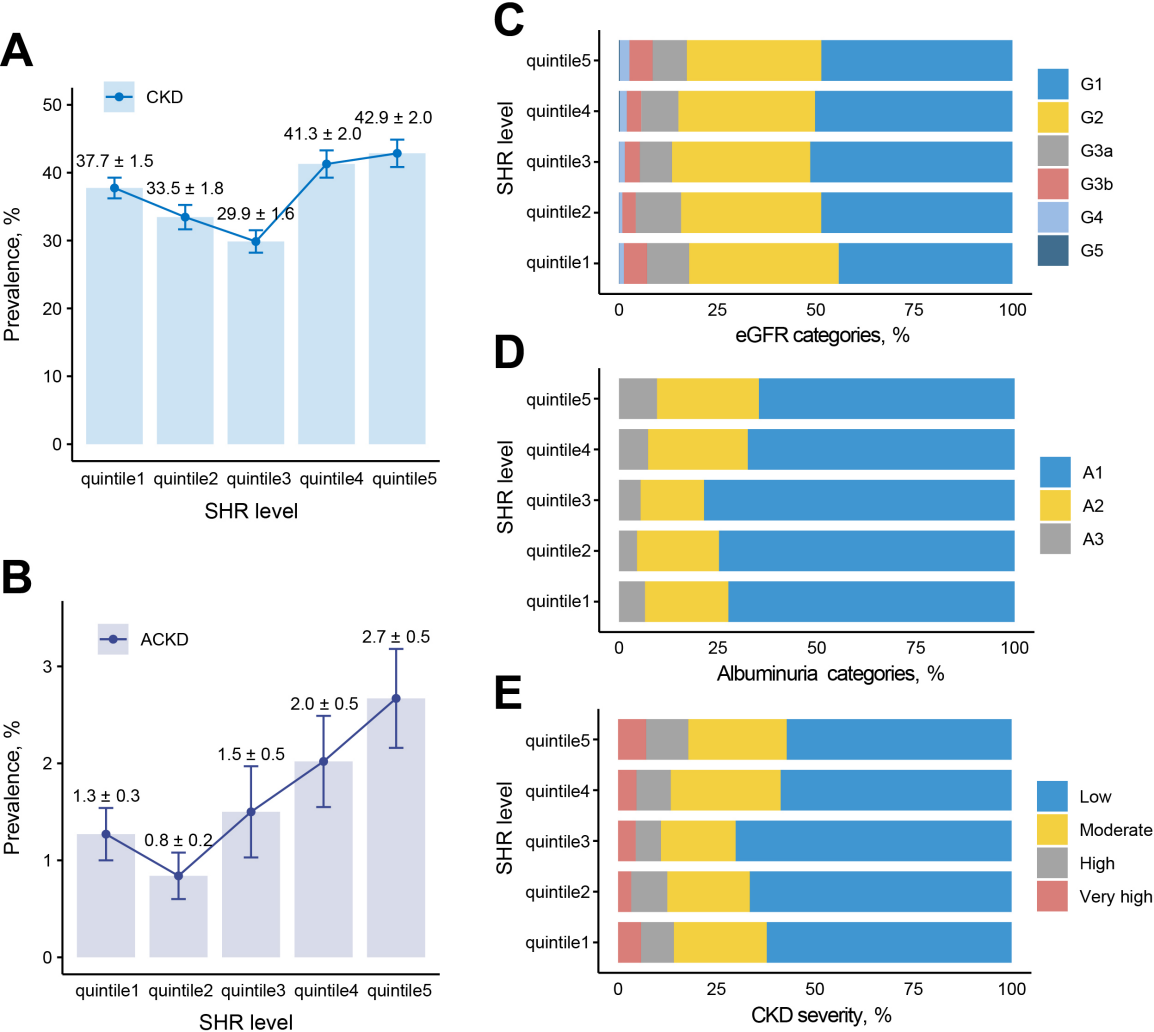
Prevalence of adverse renal events among different SHR groups. **(A)** Prevalence of CKD among 5 groups. **(B)** Prevalence of ACKD among 5 groups. **(C)** eGFR categories among 5 groups. **(D)** Albuminuria categories among 5 groups. **(E)** CKD severity among 5 groups. G1: eGFR>90 ml/min/1.73 m^2^, G2: eGFR 60-89 ml/min/1.73 m^2^, G3a: eGFR 45-59 ml/min/1.73 m^2^, G3b: eGFR 30-44 ml/min/1.73 m^2^, G4: eGFR 15-29 ml/min/1.73 m^2^, G5: eGFR <15 ml/min/1.73 m^2^; A1: uACR<30 mg/g, A2: uACR 30-300 mg/g, A3: uACR >300 mg/g; low risk: G1, G2, and A1, moderate risk: [G3a and A1] or [G1, G2 and A2], high risk: [G3b and A1], [G3a and A2], or [G1, G2 and A3], very high risk: [G4, G5 and A1], [G3b, G4, G5 and A2], or [G3a, G3b, G4, G5 and A3]. ACKD, advanced chronic kidney disease; CKD, chronic kidney disease; eGFR, estimated glomerular filtration rate; SHR, stress hyperglycemia ratio; uACR, urea albumin creatinine ratio.

Among patients with DM, the prevalence of CKD was 37.04% (SHR quintile 1 to 5 = 37.74%: 33.45%: 29.87%: 41.28%: 42.85%, P<0.001), and ACKD is 1.66% (SHR quintile 1 to 5 = 1.27%: 0.84%: 1.50%: 2.02%: 2.67%, P=0.020). Totally 23.30% patients at moderate CKD severity (SHR quintile 1 to 5 = 23.59%: 20.97%: 18.99%: 27.92%: 25.02%, P<0.001), 8.66% patients at high CKD severity (SHR quintile 1 to 5 = 8.34%: 9.15%: 6.40%: 8.72%: 10.71%, P<0.001), and 5.08% patients at very high CKD severity (SHR quintile 1 to 5 = 5.81%: 3.33%: 4.48%: 4.64%: 7.12%, P<0.001) (**Figure 2**).

**Figure 3:**
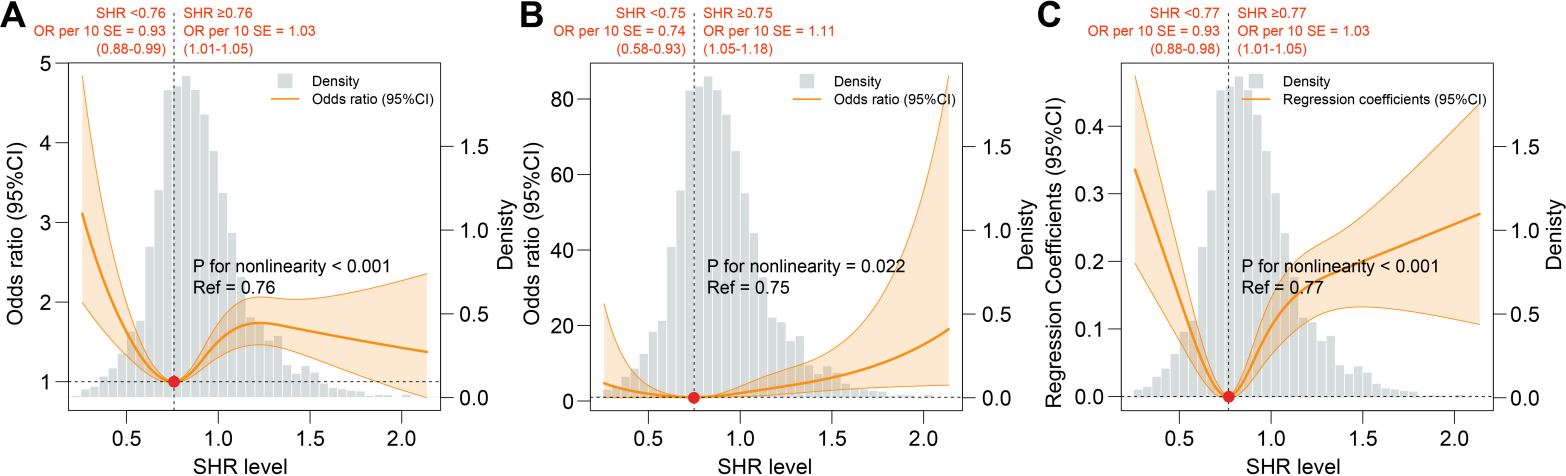
Association between SHR and adverse renal events. **(A)** Association between SHR and CKD. **(B)** Association between SHR and ACKD. **(C)** Association between SHR and CKD severity. All RCS analysis were adjusted for age, gender, body mass index, race, smoking status, alcohol consumption, DM duration, hypertension, cardiovascular disease, anemia, uric acid, blood urea nitrogen, antidiabetic drugs, renin angiotensin system inhibitors, and statins. ACKD, advanced chronic kidney disease; CKD, chronic kidney disease; SHR, stress hyperglycemia ratio.

**Figure 4:**
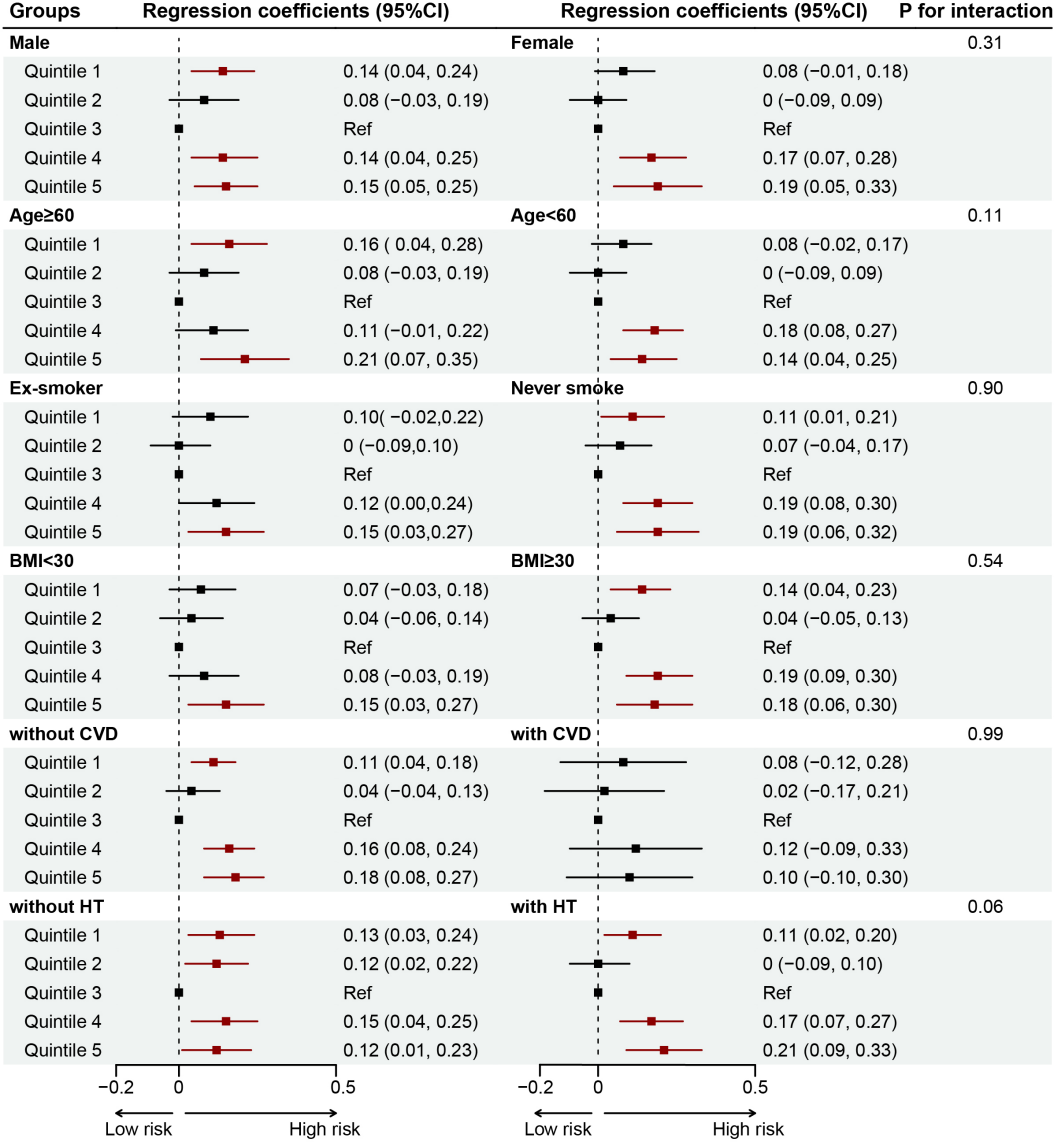
Forest plot for the associations of SHR and CKD severity in different subgroups among patients with DM. Adjusted for age, gender, body mass index, race, smoking status, alcohol consumption, DM duration, hypertension, cardiovascular disease, anemia, uric acid, blood urea nitrogen, antidiabetic drugs, renin angiotensin system inhibitors, and statins. BMI, body mass index; CVD, cardiovascular disease; HT,  hypertension.

Results of RCS analyses showed a U-shaped association between SHR and CKD (reference value = 0.76, P for nonlinearity <0.001), ACKD (reference value = 0.75, P for nonlinearity = 0.022), and CKD severity (reference value = 0.77, P for nonlinearity <0.001). Moreover, for each 10 additional standard error (SE) of SHR, the OR of CKD was 0.93 (0.88-0.99, P=0.03) when SHR was <0.76, and 1.03 (1.01-1.05, P=0.005) when SHR was ≥0.76; the OR of ACKD was 0.74 (0.58-0.93, P=0.01) when SHR was <0.75, and 1.11 (1.05-1.18, P<0.001) when SHR was ≥0.75; the OR of CKD severity was 0.93 (0.88-0.98, P=0.009) when SHR was <0.77, and 1.03 (1.01-1.05, P<0.001) when SHR was ≥0.77. (**Table 3**, **Figure 3**). Results of subgroup analysis also showed a U-shaped association between SHR and CKD severity (**Figure 4**).

**Table 3:** Impact of SHR on adverse renal outcomes.

Outcomes	Groups	Model 1		Model 2		Model 3	
		OR (95%CI)	P value	OR (95%CI)	P value	OR (95%CI)	P value
**CKD**	Quintile 1	**1.42 (1.17-1.73)**	**<0.001**	**1.54 (1.24-1.92)**	**<0.001**	**1.50 (1.19-1.90)**	**<0.001**
	Quintile 2	1.18 (0.94-1.48)	0.150	1.24 (0.97-1.58)	0.090	1.23 (0.93-1.61)	0.140
	Quintile 3	Ref	-	Ref	-	Ref	-
	Quintile 4	**1.65 (1.33-2.06)**	**<0.001**	**1.84 (1.44-2.35)**	**<0.001**	**1.95 (1.49-2.55)**	**<0.001**
	Quintile 5	**1.76 (1.38-2.24)**	**<0.001**	**1.86 (1.43-2.41)**	**<0.001**	**1.79 (1.33-2.41)**	**<0.001**
**ACKD**	Quintile 1	0.85 (0.43-1.65)	0.620	1.18 (0.57-2.48)	0.650	1.46 (0.59-3.61)	0.410
	Quintile 2	0.56 (0.24-1.32)	0.180	0.83 (0.35-1.95)	0.660	1.07 (0.42-2.72)	0.890
	Quintile 3	Ref	-	Ref	-	Ref	-
	Quintile 4	1.35 (0.62-2.93)	0.440	**2.50 (1.16-5.39)**	**0.02**	**3.28 (1.16-9.31)**	**0.030**
	Quintile 5	1.80 (0.85-3.80)	0.120	**3.50 (1.71-7.16)**	**<0.001**	**3.89 (1.64-9.25)**	**0.002**
**CKD severity**	Quintile 1	**1.42 (1.17-1.71)**	**<0.001**	**1.52 (1.24-1.87)**	**<0.001**	**1.46 (1.17-1.82)**	**<0.001**
	Quintile 2	1.17 (0.94-1.45)	0.152	1.23 (0.98-1.56)	0.077	1.20 (0.93-1.55)	0.163
	Quintile 3	Ref	-	Ref	-	Ref	-
	Quintile 4	**1.57 (1.28-1.92)**	**<0.001**	**1.76 (1.40-2.22)**	**<0.001**	**1.84 (1.42-2.39)**	**<0.001**
	Quintile 5	**1.78 (1.41-2.24)**	**<0.001**	**1.92 (1.49-2.49)**	**<0.001**	**1.83 (1.38-2.44)**	**<0.001**

Model 1 was unadjusted. Model 2 was adjusted for age, gender, body mass index, race, smoking status and alcohol consumption. Model 3 was adjusted for age, gender, body mass index, race, smoking status, alcohol consumption, DM duration, hypertension, cardiovascular disease, anemia, uric acid, blood urea nitrogen, antidiabetic drugs, renin angiotensin system inhibitors, and statins.

ACKD, advanced chronic kidney disease; CKD, chronic kidney disease; SHR, stress hyperglycemia ratio.

The bold values indicate a statistical difference with a P-value of less than 0.05.

After adjusting for confounders, compared with subjects in quintile 3, the multivariable-adjusted OR for CKD is 1.50 for quintile 1 (P<0.001), 1.23 for quintile 2 (P=0.140), 1.95 for quintile 4 (P<0.001), and 1.79 for quintile 5 (P<0.001). For ACKD, compared with quintile 3, the adjusted OR is 1.46 for quintile 1 (P=0.410), 1.07 for quintile 2 (P=0.890), 3.28 for quintile 4 (P=0.030), and 3.89 for quintile 5 (P=0.002). For CKD severity, compared with quintile 3, the adjusted OR is 1.82 for quintile 1 (P<0.001), 1.55 for quintile 2 (P=0.163), 1.84 for quintile 4 (P<0.001), and 1.83 for quintile 5 (P<0.001). Similar results were observed in secondary outcomes.

## Discussion

To our knowledge, this study is the first to assess the association between SHR and adverse renal outcomes (CKD, ACKD, and CKD severity) among the community DM population. Our findings demonstrated a U-shaped association between SHR and adverse renal outcomes, meaning that patients with too high or too low SHR level is associated with a higher risk of CKD, ACKD, and CKD severity. Similar trends were observed in different subgroups.

Recent studies have proved that SHR is related to poor prognosis or adverse cardiovascular events in patients with acute coronary syndrome, ischemic stroke, or critical illness (18, 23, 28–30). Among patients with DM or prediabetes, Ding et al. reported a U- shaped or L-shaped association with mortality in the NHANES database, which means that too high or too low has adverse effects on the prognosis (22). For renal outcomes, SHR was also demonstrated to be an independent predictor of contrast-induced acute kidney injury in patients undergoing coronary angiography or percutaneous coronary intervention (31). Among type 2 DM patients combined with heart failure, Zhou et al. found too high or too low SHR was at increased risk of major acute kidney injury (32). In addition, Yan et al. also found a U-shaped association between SHR and diabetic kidney disease (33). However, these studies did not discuss SHR levels with the CKD progression and its severity, and the results of our study showed a U-shaped association between SHR and CKD, ACKD, and CKD severity, which is consistent with the trend of previous studies, and confirm that too high or too low SHR can have adverse effects on patients’ renal function.

Stress hyperglycemia reflects an acute change in blood glucose levels caused by stress reaction or severe disease (34). For patients with DM, the effect of SHR on renal disease progression may largely attributed to the inflammatory response caused by fluctuations in blood glucose (35). Blood glucose increase can lead to an overproduction of reactive oxygen species in the endothelial cells of renal blood vessels (33). In addition, oxidative stress caused by inflammation and DM can further result in endothelial dysfunction and impaired vasodilation. Similarly, low SHR reflects episodes of hypoglycemia, which may be due to the incorrect use of insulin or antidiabetic drugs, prolonged fasting or digestive difficulties. When patients at low SHR status, hypoglycemia can also induce increased platelet activity through the elevated markers of inflammatory and oxidative stress, while causing the activation of the sympathetic adrenal system, and further leading to hemodynamic changes (36, 37).

In the current study, we found a U-shaped association between SHR for CKD and its severity in the community DM population, and the results were also stable in different subgroups. Results suggested that regularly systematic assessment of stress hyperglycemia in primary care and community health practice is necessary to preserve the most favorable levels of blood glucose. For community doctors, in addition to monitoring the SHR level of patients with DM, the control of other risk factors is also the key to reducing the occurrence of adverse renal events for patients. Further studies are needed to determine the mechanisms by which stress hyperglycemia levels affect adverse renal outcomes in DM patients.

### Limitation

There were several limitations in our study. Firstly, the data only included US citizens, therefore, the results may not apply to other countries or regions. Prospective multicenter studies are needed to further test this relationship and the underlying mechanisms involved. Secondly, due to the limitations of NHANES, we did not exclude patients with a history of acute kidney injury or preeclampsia with acute kidney injury, which may have an impact on the relationship between SHR and adverse renal outcomes. In addition, due to the nature of the cross-sectional study, this study lacks data on dynamic changes in patients’ kidney function. Finally, types of DM were not differentiated in NHANES, making it difficult to delineate between type 1 DM and type 2 DM (3).

### Conclusion

In our study, SHR shows a U-shaped association with CKD, ACKD, and CKD severity among patients with DM, and it can be an independent predictor for adverse renal outcomes. Further multicenter prospective studies are required to investigate the predictive value of SHR on dynamic changes in renal function and to control blood glucose based on SHR to improve patients’ quality of life.

## Data Availability

Publicly available datasets were analyzed in this study. This data can be found here: All data are available as publicly accessible datasets through NHANES. It is open and publicly accessible through the following link: https://wwwn.cdc.gov/nchs/nhanes/.
