# Supplementary material for: Association of stress hyperglycemia ratio with presence and severity of chronic kidney disease among US adults with diabetes mellitus
Source: Front Endocrinol (Lausanne). 2024 Oct 22;15:1446390. doi: 10.3389/fendo.2024.1446390 (PMC11534732; doi:10.3389/fendo.2024.1446390)
Supplement: Supplementary file 1 [file DataSheet1.docx]

**Supplemental Table 1. Impact of SHR on eGFR categories and albuminuria categories**

| **Outcomes** | **Groups** | **Model 1** | | **Model 2** | | **Model 3** | |
| --- | --- | --- | --- | --- | --- | --- | --- |
|  |  | **OR (95%CI)** | **P value** | **OR (95%CI)** | **P value** | **OR (95%CI)** | **P value** |
| **eGFR categories** | Quintile 1 | **1.34 (1.13-1.60)** | **<0.001** | **1.50 (1.18-1.90)** | **<0.001** | **1.52 (1.18-1.97)** | **0.001** |
|  | Quintile 2 | 1.12 (0.92-1.37) | 0.253 | 1.18 (0.94-1.47) | 0.158 | 1.09 (0.86-1.38) | 0.495 |
|  | Quintile 3 | Ref | - | Ref | - | Ref | - |
|  | Quintile 4 | 1.07 (0.87-1.31) | 0.520 | 1.21 (0.94-1.55) | 0.139 | **1.28 (1.00-1.65)** | **0.050** |
|  | Quintile 5 | 1.18 (0.97-1.44) | 0.095 | **1.35 (1.05-1.73)** | **0.020** | **1.33 (1.03-1.73)** | **0.028** |
| **Albuminuria  categories** | Quintile 1 | **1.38 (1.12-1.71)** | **0.002** | **1.45 (1.15-1.82)** | **0.002** | **1.34 (1.05-1.72)** | **0.019** |
|  | Quintile 2 | 1.20 (0.94-1.53) | 0.134 | 1.23 (0.94-1.59) | 0.129 | 1.19 (0.90-1.58) | 0.210 |
|  | Quintile 3 | Ref | - | Ref | - | Ref | - |
|  | Quintile 4 | **1.73 (1.36-2.20)** | **<0.001** | **1.79 (1.37-2.33)** | **<0.001** | **1.76 (1.32-2.35)** | **<0.001** |
|  | Quintile 5 | **2.00 (1.55-2.58)** | **<0.001** | **2.01 (1.53-2.66)** | **<0.001** | **1.85 (1.37-2.51)** | **<0.001** |

Model 1 was unadjusted. Model 2 was adjusted for age, gender, body mass index, race, smoking status and alcohol consumption. Model 3 was adjusted for age, gender, body mass index, race, smoking status, alcohol consumption, DM duration, hypertension, cardiovascular disease, anemia, uric acid, blood urea nitrogen, antidiabetic drugs, renin angiotensin system inhibitors, and statins.

The eGFR categories were G1 (eGFR>90 ml/min/1.73 m^2^), G2 (eGFR 60-89 ml/min/1.73 m^2^), G3a (eGFR 45-59 ml/min/1.73 m^2^), G3b (eGFR 30-44 ml/min/1.73 m^2^), G4 (eGFR 15-29 ml/min/1.73 m^2^) and G5 (eGFR <15 ml/min/1.73 m^2^). Albuminuria categories were A1 (uACR<30 mg/g), A2 (uACR 30-300 mg/g) and A3 (uACR >300 mg/g).

Abbreviation: ACKD = advanced chronic kidney disease, CKD = chronic kidney disease; SHR = stress hyperglycemia ratio


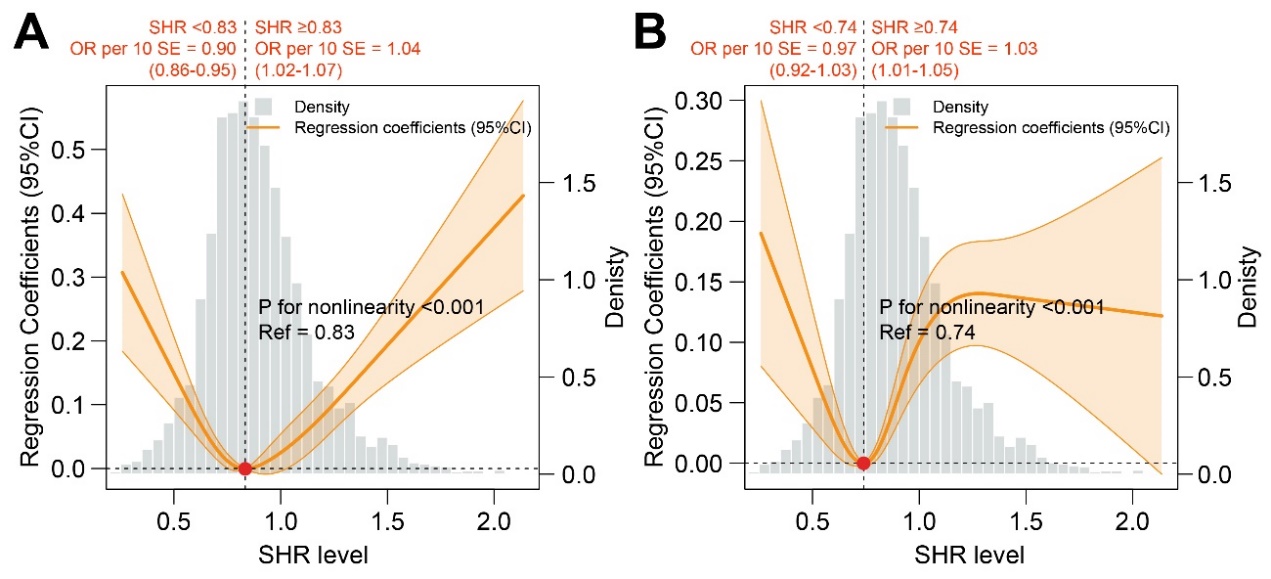
**Supplemental Figure 1. Association between SHR and eGFR categories and** **albuminuria categories among patients with DM.**

(A) Association between SHR and eGFR categories. (B) Association between SHR and albuminuria categories. Both RCS analysis was adjusted for age, gender, body mass index, race, smoking status, alcohol consumption, DM duration, hypertension, cardiovascular disease, anemia, uric acid, blood urea nitrogen, antidiabetic drugs, renin angiotensin system inhibitors, and statins.

Abbreviation: eGFR = estimated glomerular filtration rate, SHR = stress hyperglycemia ratio.
